# Supplementary material for: AAV2.7m8 is a powerful viral vector for inner ear gene therapy
Source: Nat Commun. 2019 Jan 25;10:427. doi: 10.1038/s41467-018-08243-1 (PMC6347594; doi:10.1038/s41467-018-08243-1)
Supplement: Supplementary file 1 — Supplementary Information [file 41467_2018_8243_MOESM1_ESM.docx]

**AAV2.7m8 is a powerful viral vector for inner ear gene therapy**

**Supplementary file**

**Supplementary Figure 1**

**
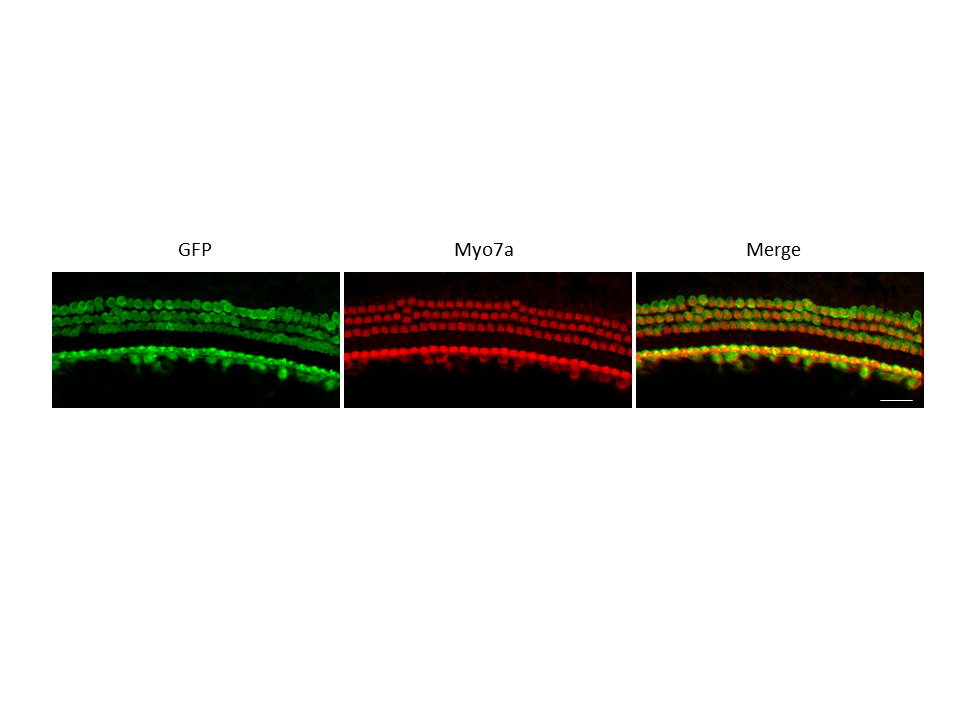
**

**Supplementary Figure 1:** **AAV2.7m8 infects adult mouse cochlear hair cells with high efficiency.** Confocal images of the cochlear middle turn from an adult CBA/J mouse (6 month old) injected with AAV2.7m8-GFP via the round window approach. Robust GFP expression is seen in both IHCs and OHCs, indicating high infection efficiency. GFP expression is shown in green, and Myo7a expression (a marker for hair cells) is shown in red. Scale bar represents 20 μm.

**Supplementary Figure 2**

**
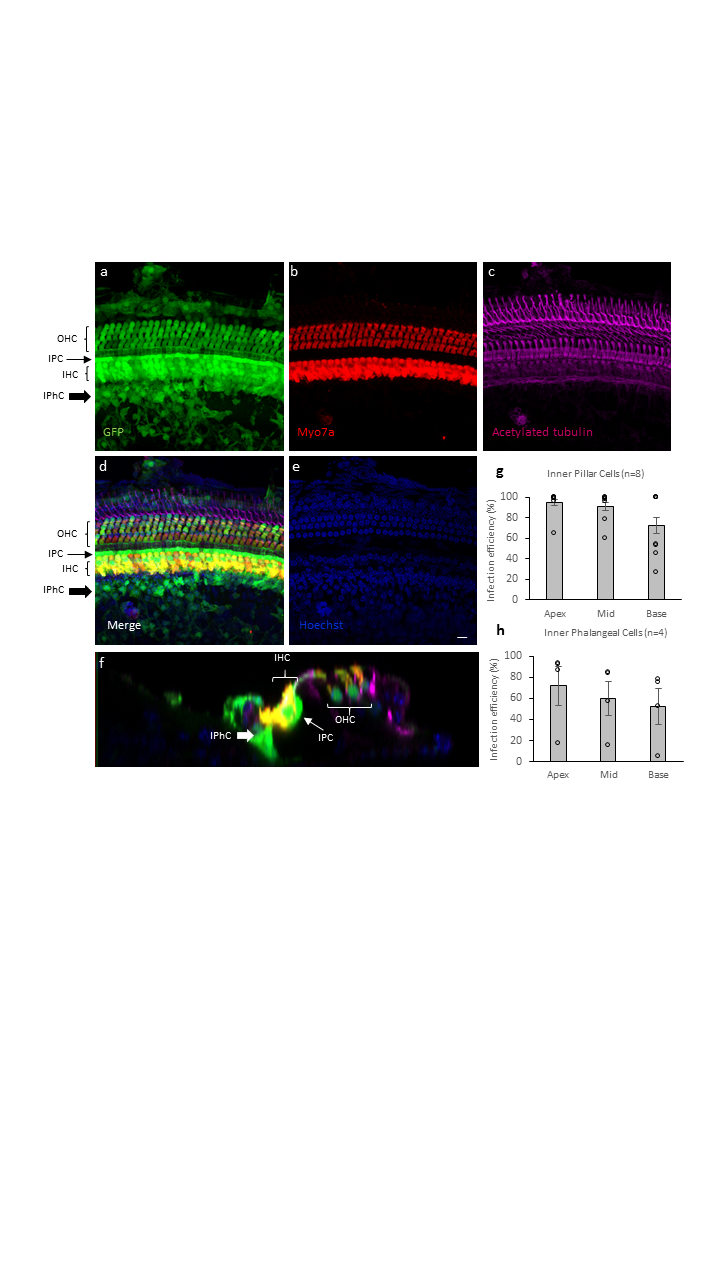
**

**Supplementary Figure 2:** **AAV2.7m8 infects inner pillar cells and inner phalangeal cells with high efficiency. (a-e)** Representative whole mount images of the cochlear apex from a mouse that underwent AAV2.7m8-GFP injection via the posterior semicircular canal approach. The inner pillar cells and inner phalangeal cells showed high levels of GFP expression. GFP expression is shown in green, Myo7a expression (a marker for hair cells) is shown in red, acetylated tubulin expression is shown in magenta (a marker for supporting cells), and Hoechst stain is shown in blue (marker for nuclei). 40x images are shown. Scale bar represents 10 μm. **(f)** Orthogonal projection of the same image showing inner pillar cells and inner phalangeal cells with robust GFP expression. Images of the cochlear apex are shown. **(g & h)** Quantification of inner pillar cell **(g)** and inner phalangeal cell **(h)** infection efficiency. Error bars represent standard errors. Open circles represent average infection efficiency of each animal. n represents the number of animals tested. Source data are provided as a Source Data file. IHC: inner hair cell. OHC: outer hair cell. IPC: inner pillar cell. IPhC: inner phalangeal cell.

**Supplementary Figure 3**

**
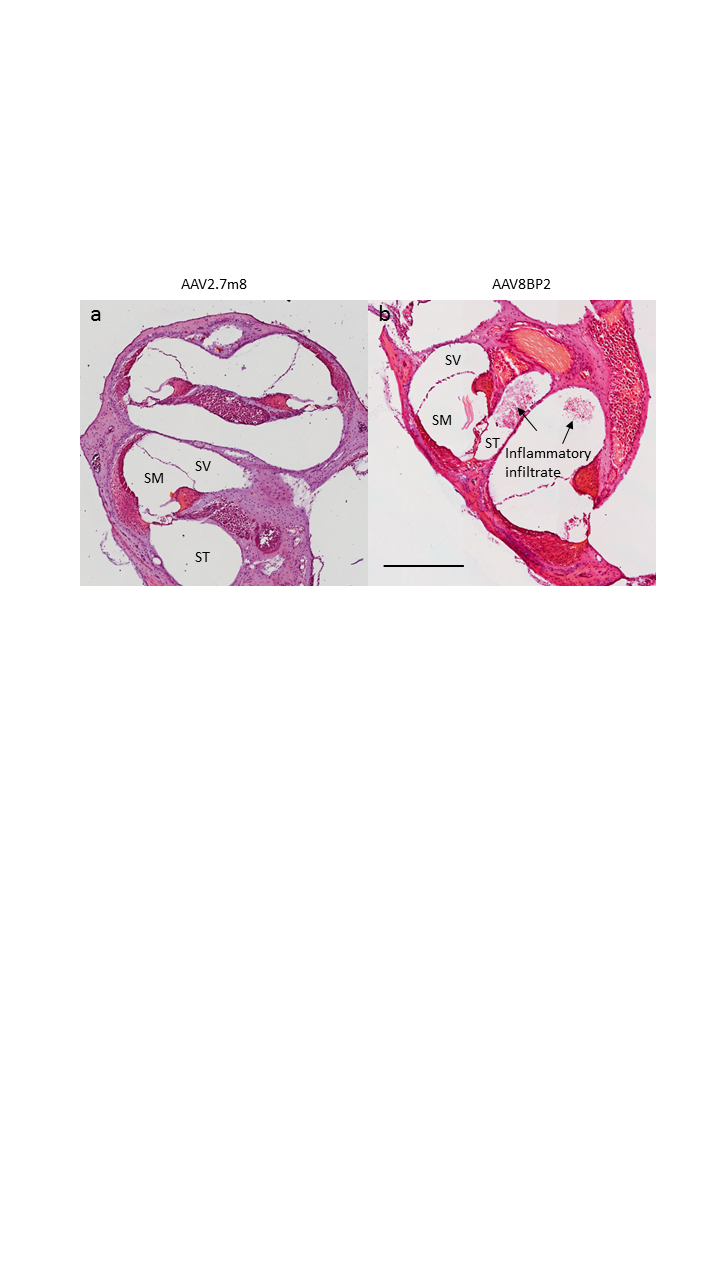
**

**Supplementary Figure 3: AAV8BP2 causes inflammation in the cochlea.** (**a**) Examination of the cochlea after AAV2.7m8-GFP injection using hematoxylin and eosin (H&E) stain showed no evidence of inflammatory cell infiltration. (**b**) In contrast, infiltration of inflammatory cells was seen in the cochlea after AAV8BP2 injection. SV: scala vestibuli. SM: scala media. ST: scala tympani. Scale bar represents 250 μm.

**Supplementary Figure 4**

**
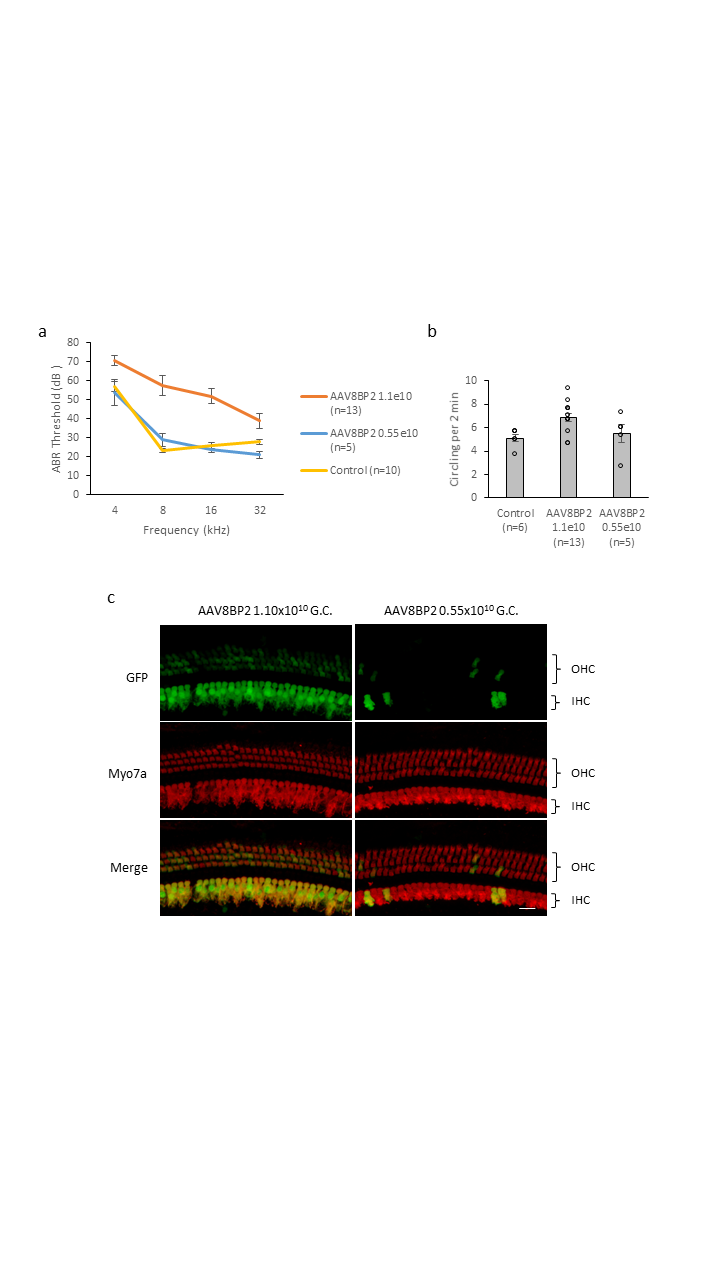
**

**Supplementary Figure 4: AAV8BP2 does not cause hearing loss and increased circling at lower concentration. (a)** Posterior canal injection of AAV8BP2 at 0.55x10^10^ genome copies (GC) (AAV8BP2 0.55e10) caused no ABR threshold elevation compared to non-injected control mice. (**b**) Posterior canal injection of AAV8BP2 at 0.55x10^10^ GC (AAV8BP2 0.55e10) caused no elevation in circling behavior compared to non-injected control mice. Error bars represent standard errors. Open circles represent average value of each animal. n represents the number of animals tested. Source data are provided as a Source Data file. (**c**) The infection efficiency of IHCs and OHCs is lower when AAV8BP2 is delivered at 0.55x10^10^ GC compared to 1.10x10^10^ GC Images taken from cochlear apex. Scale bar represents 20 μm.

**Supplementary Table 1**

**Viral infection efficiency**

|  | **IHC** | **OHC** | **Utricle** | **IPC** | **IPhC** |
| --- | --- | --- | --- | --- | --- |
| **AAV2.7m8** | 84.1 (5.66) | 83.1 (6.17) | 27.5 (9.65) | 86.1 (4.56) | 61.4 (9.30) |
| **AAV8BP2** | 55.7 (9.53) | 44.1 (7.94) | 34.2 (9.84) | 0  (0) | 0  (0) |
| **AAV2** | 43.6 (13.5) | 54.5 (12.7) | 32.4 (6.16) | 60.3 (7.96) | 0  (0) |
| **AAV8** | 86.0 (5.34) | 51.7 (5.95) | 93.3 (2.15) | 50.4 (7.49) | 0  (0) |
| **Anc80L65** | 94.0 (3.63) | 67.0 (4.32) | 67.7 (2.46) | 75.3 (4.94) | 0  (0) |

Infection efficiency of AAVs in various cell types in the inner ear. The infection rate (%) as well as the standard error (in parenthesis) are shown. IHC: inner hair cell. OHC: outer hair cell. IPC: inner pillar cell. IPhC: inner phalangeal cell.
