# Supplementary material for: AAV2.7m8 is a powerful viral vector for inner ear gene therapy
Source: Nat Commun. 2019 Jan 25;10:427. doi: 10.1038/s41467-018-08243-1 (PMC6347594; doi:10.1038/s41467-018-08243-1)
Supplement: Supplementary file 3 — Reporting Summary [file 41467_2018_8243_MOESM3_ESM.pdf]

natureresearch

Corresponding author(s): Wade Chien
Last updated by author(s): Dec 9, 2018

Reporting Summary

Nature Research wishes to improve the reproducibility of the work that we publish. This form provides structure for consistency and transparency in reporting. For further information on Nature Research policies, see Authors & Referees and the Editorial Policy Checklist.

Statistics

For all statistical analyses, confirm that the following items are present in the figure legend, table legend, main text, or Methods section.

n/a	Confirmed	
☐	☒	The exact sample size (*n*) for each experimental group/condition, given as a discrete number and unit of measurement
☐	☒	A statement on whether measurements were taken from distinct samples or whether the same sample was measured repeatedly
☐	☒	The statistical test(s) used AND whether they are one- or two-sided<br>*Only common tests should be described solely by name; describe more complex techniques in the Methods section.*
☒	☐	A description of all covariates tested
☒	☐	A description of any assumptions or corrections, such as tests of normality and adjustment for multiple comparisons
☐	☒	A full description of the statistical parameters including central tendency (e.g. means) or other basic estimates (e.g. regression coefficient) AND variation (e.g. standard deviation) or associated estimates of uncertainty (e.g. confidence intervals)
☐	☒	For null hypothesis testing, the test statistic (e.g. *F*, *t*, *r*) with confidence intervals, effect sizes, degrees of freedom and *P* value noted<br>*Give P values as exact values whenever suitable.*
☒	☐	For Bayesian analysis, information on the choice of priors and Markov chain Monte Carlo settings
☒	☐	For hierarchical and complex designs, identification of the appropriate level for tests and full reporting of outcomes
☒	☐	Estimates of effect sizes (e.g. Cohen's *d*, Pearson's *r*), indicating how they were calculated

Our web collection on statistics for biologists contains articles on many of the points above.

Software and code

Policy information about availability of computer code

Data collection	No custom computer codes were used in this study.
Data analysis	Microsoft Excel and STATA were used for data analysis.

For manuscripts utilizing custom algorithms or software that are central to the research but not yet described in published literature, software must be made available to editors/reviewers. We strongly encourage code deposition in a community repository (e.g. GitHub). See the Nature Research guidelines for submitting code & software for further information.

Data

Policy information about availability of data

All manuscripts must include a data availability statement. This statement should provide the following information, where applicable:
- Accession codes, unique identifiers, or web links for publicly available datasets
- A list of figures that have associated raw data
- A description of any restrictions on data availability

A data availability statement is included in the text.

Field-specific reporting

Please select the one below that is the best fit for your research. If you are not sure, read the appropriate sections before making your selection.

☒ Life sciences ☐ Behavioural & social sciences ☐ Ecological, evolutionary & environmental sciences

For a reference copy of the document with all sections, see nature.com/documents/nr-reporting-summary-flat.pdf

Life sciences study design

All studies must disclose on these points even when the disclosure is negative.

Sample size	Sample size was determined by using data from prior similar studies.
Data exclusions	Data was excluded form mice that showed no transduction in any inner ear cell type as this indicated a technical error in inner ear gene delivery.
Replication	All experiments were performed in at least three animals to verify reproducibility.
Randomization	Each litter of pups was randomly assigned to receive a specific AAV serotype.
Blinding	Data analysis for viral infection efficiency was performed in a blinded manner. Circling behavior was calculated using a computer software.

Reporting for specific materials, systems and methods

We require information from authors about some types of materials, experimental systems and methods used in many studies. Here, indicate whether each material, system or method listed is relevant to your study. If you are not sure if a list item applies to your research, read the appropriate section before selecting a response.

Materials & experimental systems

n/a	Involved in the study	
☐	☒	Antibodies
☒	☐	Eukaryotic cell lines
☒	☐	Palaeontology
☐	☒	Animals and other organisms
☒	☐	Human research participants
☒	☐	Clinical data

Methods

n/a	Involved in the study	
☒	☐	ChIP-seq
☒	☐	Flow cytometry
☒	☐	MRI-based neuroimaging

Antibodies

Antibodies used	1. Mouse anti-myosin 7a antibody to label hair cells (1:200, product #25-6790, Proteus BioSciences, Ramona, CA).<br>2. Mouse anti-acetylated tubulin antibody to label supporting cells (1:100, product #T9026, Sigma-Aldrich Corp., St. Louis, MO).<br>3. Chicken anti-GFP antibody (1:1000, product # ab13970, Abcam, Cambridge, MA).
Validation	1. http://www.proteus-biosciences.com/Product/View/Myosin-VIIa-248.aspx<br>2. https://www.sigmaaldrich.com/catalog/product/sigma/t9026?lang=en&region=US<br>3. https://www.abcam.com/gfp-antibody-ab13970.html

Animals and other organisms

Policy information about studies involving animals; ARRIVE guidelines recommended for reporting animal research

Laboratory animals	Mouse, CBA/J
Wild animals	N/A
Field-collected samples	N/A
Ethics oversight	Animal surgery was approved by the Animal Care and Use Committee at the National Institute on Deafness and Other Communication Disorders (NIDCD ASP1378-18).

Note that full information on the approval of the study protocol must also be provided in the manuscript.
